# Cytotoxicity, Antioxidant, Antibacterial, and Photocatalytic Activities of ZnO–CdS Powders

**DOI:** 10.3390/ma13010182

**Published:** 2020-01-02

**Authors:** Irina Zgura, Nicoleta Preda, Monica Enculescu, Lucian Diamandescu, Catalin Negrila, Mihaela Bacalum, Camelia Ungureanu, Marcela Elisabeta Barbinta-Patrascu

**Affiliations:** 1 National Institute of Materials Physics, Atomistilor 405A, 077125 Magurele, Romania; mdatcu@infim.ro (M.E.); ldiamandescu@gmail.com (L.D.); catalin.negrila@infim.ro (C.N.); 2 Department of Life and Environmental Physics, Horia Hulubei National Institute for Physics and Nuclear Engineering (IFIN-HH), Bucharest, 077125 Magurele, Romania; bmihaela@nipne.ro; 3 Faculty of Applied Chemistry and Materials Science, University “Politehnica” of Bucharest, 1-7, Polizu Street, 011061 Bucharest, Romania; ungureanucamelia@gmail.com; 4 Faculty of Physics, University of Bucharest, 405 Atomistilor Street, PO Box MG-11, Bucharest, 077125 Magurele, Romania; elipatras@gmail.com

**Keywords:** ZnO–CdS composites, methylene blue discoloration, antioxidant capacity, antibacterial activity, cytotoxicity

## Abstract

In this work, ZnO–CdS composite powders synthesized by a simple chemical precipitation method were thoroughly characterized. The morphological, structural, compositional, photocatalytical, and biological properties of the prepared composites were investigated in comparison with those of the pristine components and correlated with the CdS concentration. ZnO–CdS composites contain flower-like structures, their size being tuned by the CdS amount added during the chemical synthesis. The photocatalytic activity of the composites was analyzed under UV irradiation using powders impregnated with methylene blue; the tests confirming that the presence of CdS along the ZnO in composites can improve the dye discoloration. The biological properties such as antioxidant capacity, antibacterial activity, and cytotoxicity of the ZnO, CdS, and ZnO–CdS composites were evaluated. Thus, the obtained composites presented medium antioxidant effect, biocidal activity against *Escherichia coli,* and no toxicity (at concentrations less than 0.05 mg/mL for composites with a low CdS amount) for human fibroblast cells. Based on these results, such composites can be used as photocatalytic and/or biocidal additives for photoactive coatings, paints, or epoxy floors, which in their turn can provide a cleaner and healthier environment.

## 1. Introduction

In the last years, inorganic nanostructures such as TiO_2_ [1,2], ZnO [3,4], CdS [5,6,7], Ag_2_S [8], or CdSe [9,10] have been extensively studied for their applications in the people’s daily life. Their features like size, shape, specific surface area, defects in the crystal structure, reactivity, monodispersity, etc. are responsible for the particular properties relevant in the biological area. For example, due to the size smaller than cells, the nanostructures can penetrate the biological structures disrupting their normal function [11]. Therefore, to benefit from technologies based on these low-dimensional structures, it is required to characterize them for evaluating, understanding and tuning their biological effects such as cytotoxicity, antioxidant capacity, and antibacterial activity in order to minimize or prevent adverse health impacts.

Probably the most extensively studied metal oxide, ZnO can be relatively easy prepared by various methods: Chemical precipitation [12,13], electroless deposition [14], hydrothermal [13,15,16], electrochemical deposition [17], thermal oxidation [18,19], aqueous solution growth [19], chemical vapor deposition [15], magnetron sputtering [20], and so on. This metal oxide exhibits unique properties like direct and wide band gap (~3.3 eV), high exciton binding energy (60 meV) at ambient temperature, high electrochemical stability, anisotropic growth, etc. [21]. Furthermore, being one of the best bio-friendly absorbers of UV radiation [22], ZnO can act as an antibacterial [23,24] or antifungal [25] agent when its nanostructures are incorporated in surface coatings (paints) [23,26] or textiles materials [27]. For instance, photocatalysis tests carried out recently on poly(alkyl siloxane) incorporating ZnO nanoparticles reveal that this material can be used as a protective coating of sandstone [28]. Although, for enhancing the ZnO photocatalytic efficiency in the visible range of solar energy, this semiconductor is required to be combined with a photosensitizer [29], in order to obtain a fast separation of the charge carriers and to decrease their recombination rate [30]. Hence, composite materials such as ZnO–CdS [31,32], ZnO–ZnS [33], ZnO–TiO_2_ [34], ZnO–CuO [35,36], and ZnO–Ag [37] have attracted considerable attention. CdS is considered one of the most suitable visible sensitizers, being characterized by an energy band gap value of ~2.4 eV (in bulk) and a high optical absorption coefficient. Moreover, the composites based on ZnO and CdS find applications in gas sensing [38], photocatalysis [32,39], water splitting [40], or antibacterial fields [41].

Taking into account that both semiconductors can behave as photocatalysts in the photodegradation of the environmental organic pollutants [42] and as antibacterial agents [41], their combination in composites can lead to materials with enhanced photocatalytic and antibacterial properties depending on their design and on the ratio between the two components. It has to be noticed that, recently, nanomaterials containing cadmium reveal a bactericidal effect owing to Cd^2^+ release [11,43] being used in the biological area [44,45]. However, till now, only few studies were focused on the photocatalytic and biological properties of composites based on ZnO–CdS [46,47], ZnO–CdS–Ag [48], Cd:Ag:ZnO [49], and ZnO/Cd (OH)Cl [50]. The results of these works show the huge potential of such composites in different application areas, ranging from wastewater treatment to antimicrobials. Nevertheless, the toxicity of the materials containing cadmium is still on debate. Usually, antimicrobial agents are used in the health domain for killing microorganisms or inhibiting their growth, the strength of these compounds being directly proportional to their toxicity to human cells [51]. Concerning the ZnO toxicity, there are several studies on its effect on human fibroblast cells [52]. Such researches are important because the human skin is the first organ which comes into contact with the semiconductor nanostructures. However, to our knowledge, up to now there is no report on the effect of ZnO–CdS composites on human fibroblast cells and on the antioxidant activity of these composites. 

In this context, the present work is focused on the photocatalytic activity and biological properties such as antioxidant capacity, antibacterial activity, and cytotoxicity of the ZnO, CdS, and ZnO–CdS composite powders synthesized by a simple chemical precipitation method. The properties of the prepared composites were investigated in comparison with the pristine components and correlated with the CdS concentration. The photocatalysis tests made under UV irradiation directly on powders impregnated with methylene blue confirm that the presence of CdS beside ZnO improves the dye photocatalytic discoloration. Further, the ZnO–CdS composites present a medium antioxidant effect, antibacterial activity against *Escherichia coli,* and, in the case of the composite with the lower CdS amount (at a certain concentration), no toxicity for human fibroblast cells. Ergo, the information provided by this work offers an interesting insight on the potential applications of ZnO–CdS composites as photocatalytic and/or biocidal additives for photoactive coatings, paints, epoxy floors, etc. 

## 2. Materials and Experimental Methods

The chemical reagents Cd(NO_3_)_2_·4H_2_O, Na_2_S, Zn(NO_3_)_2_·6H_2_O, and NaOH were purchased from Merck (Darmstadt, Germany) and used without further purification.

ZnO, CdS, and ZnO–CdS powders were synthesized in water, at room temperature, according to the procedure described in [53]. Thus, CdS powder was prepared by mixing 1 M Na_2_S and 1 M Cd(NO_3_)_2_·4H_2_O, the precipitate being collected by centrifugation, washed with distilled water and dried under vacuum at 100 °C for 2 h. Then, 0.5 M Zn(NO_3_)_2_ and 1 M NaOH were added in an appropriate water volume containing different CdS amounts. The obtained powder was collected by centrifugation, washed with distilled water, and dried under vacuum at 100 °C for 2 h. Additionally, for comparison, ZnO powder was obtained using the same experimental parameters. Depending on the CdS concentration (5, 10, 15, 20, and 25 mM), the ZnO–CdS composite powders were labeled as follows: ZnO–CdS5, ZnO–CdS10, ZnO–CdS15, ZnO–CdS20, and ZnO–CdS25. 

The morphological, optical, structural, compositional, photocatalytical, and biological properties of the prepared powders were evaluated. 

Scanning electron microscopy (SEM) images were obtained using a Zeiss EVO 50XVP microscope (Oberkochen, Germany). The reflectance spectra were recorded with a PerkinElmer Lambda 45 spectrometer using integrating sphere. The reflectance and photoluminescence (PL) spectra were recorded with a PerkinElmer Lambda 45 spectrometer (Waltham, MA, USA) using an integrating sphere and an Edinburgh FL 920 photoluminescence spectrometer (Livingston, UK) with double monochromators and a 450 W Xe lamp as excitation source, respectively. X-ray diffraction (XRD) analysis was carried out using a Bruker D8 Advance diffractometer (Billerica, MA, USA) with CuKα radiation. The source was operated at 40 kV and 40 mA and the Kβ radiation was eliminated using a nickel filter (λ = 0.154 nm). Diffraction patterns were acquired at room temperature in Bragg-Brentano geometry in the range of 2θ, from 20° to 80°, at a speed of 0.6°/min (2θ/min). The XRD data were processed using “Bruker Diffrac plus Basic Package Evaluation v.12”. In order to evidence the chemical states of the components, X-ray photoelectron spectroscopy (XPS) measurements were performed using a SPECS photoelectron spectrometer with a PHOIBOS 150 analyzer (Berlin, Germany). The X-ray source, XR-50, operates on an Al anode (hν = 1486.7 eV) at 300 W, 24 mA, and 12.5 kV. The acquisition was made with pass energy of 10 eV for individual spectra and 50 eV for extended spectrum. 

The photocatalytic properties were studied using a Photocatalysis Evaluation Checker model PCC-2 (ULVAC RIKO Inc., Yokohama, Kanagawa, Japan) and methylene blue as dye (MB, Merck). In a very fast and simple way, the PCC-2 system can compare in the same time the photocatalytic activities of two samples. Thus, for investigating the M over time under continuous irradiation with UV light, the device generates pulsed light at 660 nm (at this wavelength being the main absorption peak of MB) and records only the reflected impulses from the sample surface synchronized with the light emitter period. The photocatalytic measurements were performed at room temperature, under UV light (~1 mW; λ = 368 nm), at~2.5 W/m^2^ irradiation, over a period of 120 min. Firstly, droplets of the powder suspension in ethanol were dripped onto glass substrates. In order to remove any contaminants, the samples were cleaned for 2 h under a UV lamp of 30 W (λ = 365 nm). Further, they were immersed in 1 mM MB aqueous solution for 2 h and dried in dark for several days at the room temperature. In this way, the water molecules were eliminated, only the MB molecules remaining on the powder surface. During the measurements, the MB decomposition leads to a gradual reduction of its optical absorption. The system provides the absorbance value, a higher negative value indicating a better photocatalytic activity of the sample [54]. The percentage of dye discoloration was estimated using the equation *Discoloration efficiency* = (*C*_0_ − *C*)/*C*_0_ × 100%, where, *C*_0_ is the initial value of the dye concentration, *C* is the value of the dye concentration at *t* time.

The active species (hydroxyl radicals) involved in the MB discoloration were detected by photoluminescence using terephthalic acid (TA, Merck) as a probe molecule. Thus, an amount of 5 mg of powder sample was dispersed in 20 mL of 0.5 mM TA in 2 mM NaOH aqueous solution. Then, at intervals of 15 min, a volume of 3 mL of the obtained suspension was exposed to unfiltered light provided by a 50 W low pressure mercury vapor lamp. The sample was placed at 10 cm distance from the light source. Under light irradiation, the photocatalyst powders generate hydroxyl radicals (OH•), which further react with TA producing 2-hydroxyterephthalic acid (TAOH), a highly fluorescent product [55]. The emission spectra were collected at room temperature by a LS55 Perkin Elmer fluorescence spectrometer using λ_exc_ = 315 nm, in 350–600 nm wavelength range. 

The antioxidant capacity was assessed by chemiluminescence (CL) technique, on a Chemiluminometer Turner Design TD 20/20 (USA), using the free radical generator system based on luminol (1 mM), H_2_O_2_ (10 μM) in Tris–HCl buffer solution (pH 8.6), all chemicals being bought from Merck. The in vitro antioxidant activity of the investigated samples was evaluated using the equation *Antioxidant activity* = [(*I*_0_ − *I*)/*I*_0_] × 100%, where *I*_0_ is the maximum CL intensity at t = 5 s, for the reaction mixture without the sample, and *I* is the maximum CL intensity at t = 5 s, for the reaction mixture in the presence of the sample [56].

Antibacterial activity was assessed against pathogenic Gram-negative bacteria, *Escherichia coli* ATCC 8738, by agar well diffusion method (qualitative assessment) [57,58]. The bacterial strain was grown in Luria Bertani Agar (LBA) plates at 37 °C, in a medium with the following composition—20 g/L agar (Fluka), 10 g/L peptone (Merck), 5 g/L yeast extract (Biolife), and 5 g/L NaCl (Sigma Aldrich). The stock culture was maintained at 4 °C, the agar surface being inoculated by spreading a volume of the bacterium inoculums. Thus, a well (diameter of 6 mm) punched (aseptically) with a sterile cork borer was filled with a volume of 50 µL of the sample. Further, agar plates were incubated at 37 °C for 24 h. The presence of a clear zone of inhibition (ZOI, mm) after incubation indicates the antimicrobial effectiveness of the investigated sample. 

In the case of cell culture, human fibroblast BJ cells (ATCC CRL-2522, USA) were grown in MEM (minimal essential medium) supplemented with 2 mM l-Glutamine, 10% fetal calf serum (FCS), 100 units/mL of penicillin, and 100 µg/mL of streptomycin at 37 °C in a humidified incubator under an atmosphere containing 5% CO_2_. All cell cultivation media and reagents were purchased from Biochrom AG. 

The biocompatibility was analyzed using (3-(4,5-dimethylthiazol-2-yl)-2,5-diphenyltetrazolium bromide) tetrazolium, *MTT* reduction assay, the cells being seeded in 96 well plates (25,000 cells/well) and cultured for 24 h in medium. After overnight incubation, the medium was changed and the investigated sample in concentration varying from 0.03 to 0.25 mg/mL was added for 24 h. As negative control, cells in medium without the investigated samples were used. Following incubation, the medium was changed and MTT solution was added to each well to a final concentration of 1 mg/mL and incubated for an additional 4 h, at 37 °C. Finally, the medium was collected and dimethylsulfoxide (DMSO) was used to dissolve the insoluble formazan product. The absorbance of the samples was recorded at 570 nm using a plate reader Mithras 940 (Berthold, Bad Wildbad, Germany). The data were corrected for the background and the percentage of viable cells was obtained using the equation *Cell viability* = [(A570 of treated cells)/(A570 of untreated cells)] × 100%. The sample concentration that reduced the viability of the cells by half (IC50) were estimated by fitting the data with a logistical sigmoidal equation using the software Origin 8.1 (Microcal Inc., Northampton, MA, USA).

## 3. Results and Discussions

**Figure 1:**
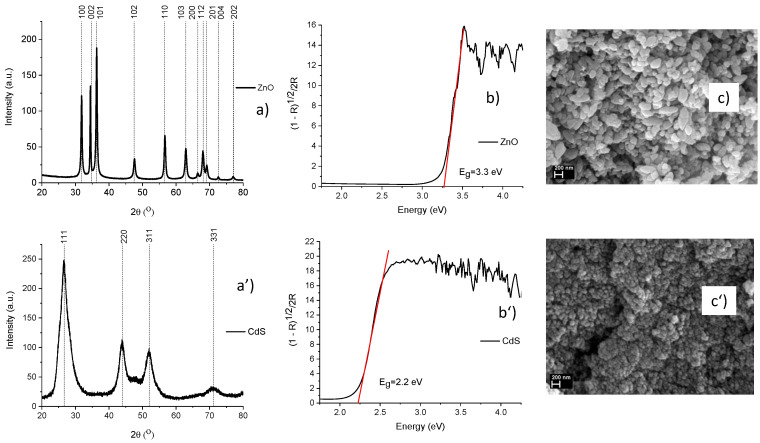
(**a**,**a’**) XRD patterns; (**b**,**b’**) the representation of the Kubelka-Munk function involved in the estimation of the band gap values; (**c**,**c’**) the SEM images of the pristine components: (**a**–**c**) ZnO and (**a’**–**c’**) CdS.

Firstly, both components of the composites, ZnO and CdS, were investigated from structural, optical, and morphological points of view (Figure 1). Thus, in the Figure 1a, the diffraction peaks at 31.8°, 34.7°, 36.3°, 47.6°, 56.6°, 62.9°, 66.4°, 67.9°, and 69.1° correspond to the Miller indices of the reflecting planes (100), (002), (101), (102), (110), (103), (200), (112), and (201), respectively, indexed to hexagonal wurtzite phase of ZnO (ICDD file no. 89-0510). In Figure 1a’, the main diffraction peaks at 26.5°, 43.9°, and 54.5° correspond to the Miller indices of the reflecting planes (111), (220), and (222), respectively, assigned to the face-centered cubic phase of CdS (ICDD file no. 89-0440). Based on the reflectance data, from the representation of Kubelka-Munk function F (R) = (1 − R)^1/2^/2R, where R is the diffuse reflectance, the band gap value was estimated to be ~3.3 eV for ZnO (Figure 1b) and ~2.2 eV for CdS (Figure 1b’); the values being in good agreement with those reported in the literature [7,18]. The SEM images of both pristine components revealed that the ZnO powder is formed by spindle structures with dimensions between 100 and 500 nm (Figure 1c), while the CdS powder contains agglomerate nanoparticles of ~50 nm (Figure 1c’). 

**Figure 2:**
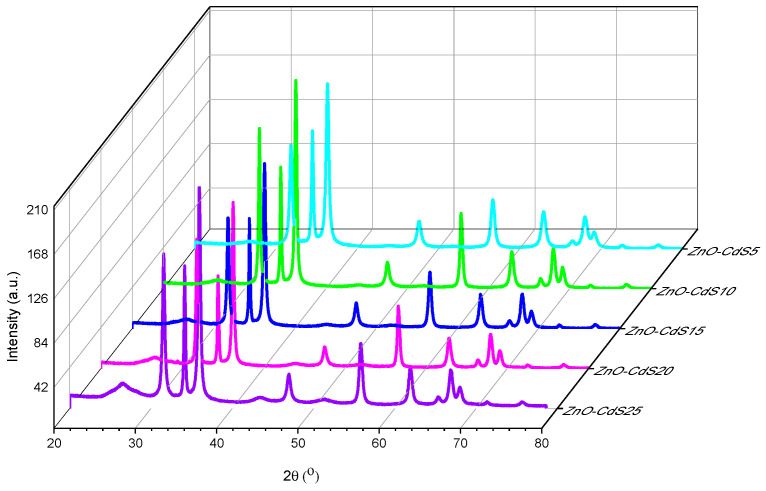
XRD patterns of ZnO–CdS powders.

In the case of ZnO–CdS composite powders, the XRD patterns exhibit mixed diffraction peaks indexed to ZnO and CdS pristine components (Figure 2). It can be seen that the intensity of the diffraction peak situated at 26.5°, corresponding to the (111) plane of CdS, became stronger with the increase of the CdS amount in the composites. 

**Figure 3:**
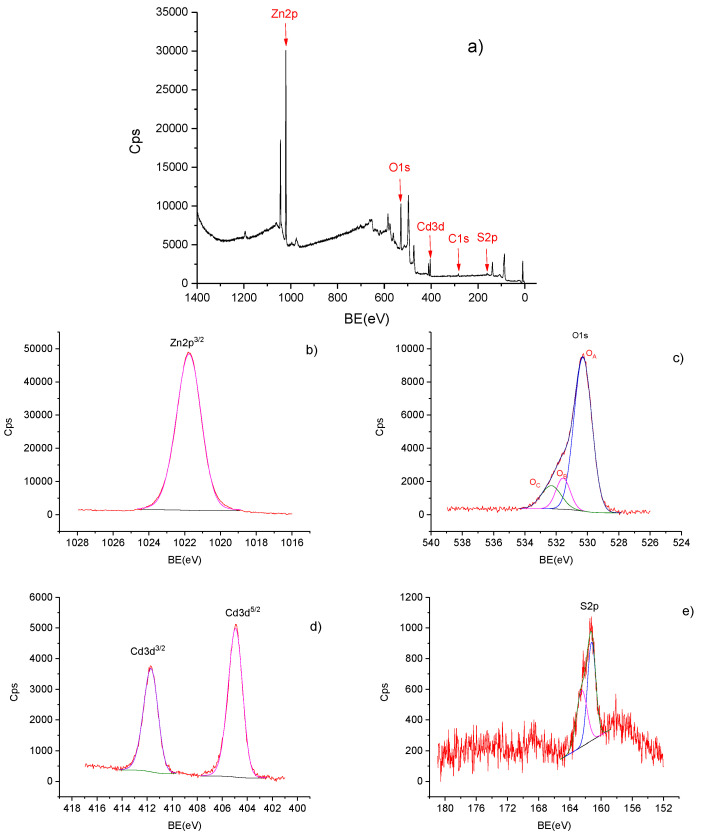
XPS spectra of ZnO–CdS15 powder: (**a**) Survey spectrum; (**b**) Zn2p^3/2^ spectrum; (**c**) O1s spectrum; (**d**) Cd3d spectrum; (**e**) S2p spectrum.

The surface chemical states of the composites were investigated by XPS analysis; Figure 3 presents the data obtained for the ZnO–CdS15 composite powder. The XPS technique allows the information on the chemical composition from the surface sample and up to 10 nm in depth to be acquired [59]. Thus, in the survey spectrum (Figure 3a), the peaks can be attributed to Zn, O, Cd, S, and C; their concentrations are given in Table 1. The signature of C is linked to the hydrocarbon contaminants, which commonly appeared in the XPS spectra [60]. In Figure 3b, the peak at 1021.76 eV is assigned to Zn2p^3/2^, confirming the presence of Zn in the Zn^2+^ chemical state [55]. In Figure 3c, the peaks at binding energies of 530.29, 531.56, and 532.31 eV are linked to the lattice oxygen of ZnO (OA) and molecular adsorbed oxygen (OB) coming from the contamination of the surface with oxygen and C–O bonds, which are usually found on the surface sample exposed to air (OC, 532.31 eV), respectively [60]. It should be noted that oxygen vacancies and chemisorbed oxygen are involved in the photocatalytic reactions by rapid charge transfer and also in the generation of the hydroxyl radicals [48]. In Figure 3d, the peaks at 404.94 and 411.69 eV are assigned to Cd3d^5/2^ and Cd3d^3/2^, respectively, being characteristic for Cd^2+^ in CdS bond [60]. In Figure 3e, the peaks at 161.22 and 162.48 eV are associated with S2p^3/2^ and S2p^1/2^, respectively, confirming that S exists mainly in the S^2−^ chemical state on the composite sample [61]. Thus, it can be concluded that the investigated composite sample contains only ZnO and CdS; similar XPS data was also recorded for the other composites. 

**Figure 4:**
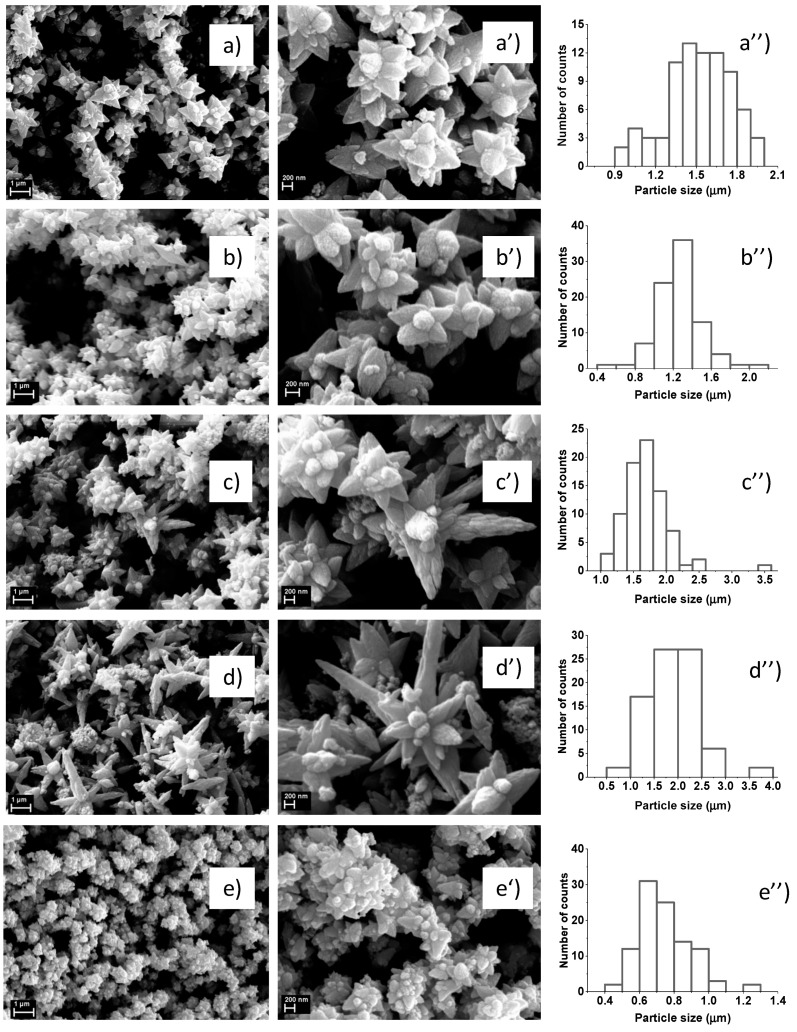
SEM images at two magnifications and particle size distribution histograms of ZnO–CdS powders: (**a**,**a’**,**a’’**) ZnO–CdS5; (**b**,**b’**,**b’’**) ZnO–CdS10; (**c**,**c’**,**c’’**) ZnO–CdS15; (**d**,**d’**,**d’’**) ZnO–CdS20; (**e**,**e’**,**e’’**) ZnO–CdS25.

The SEM images of synthesized ZnO–CdS composite powders (Figure 4) reveal a flower-like structures, with sizes depending on the CdS amount added during the chemical synthesis. The growth mechanism involved in the chemical synthesis of ZnO–CdS composites by chemical precipitation, which leads to this particular morphology, was detailed in [53]. Based on the SEM images at lower magnification and using the ImageJ software, we obtained the particle size distribution histograms for all investigated samples.

**Figure 5:**
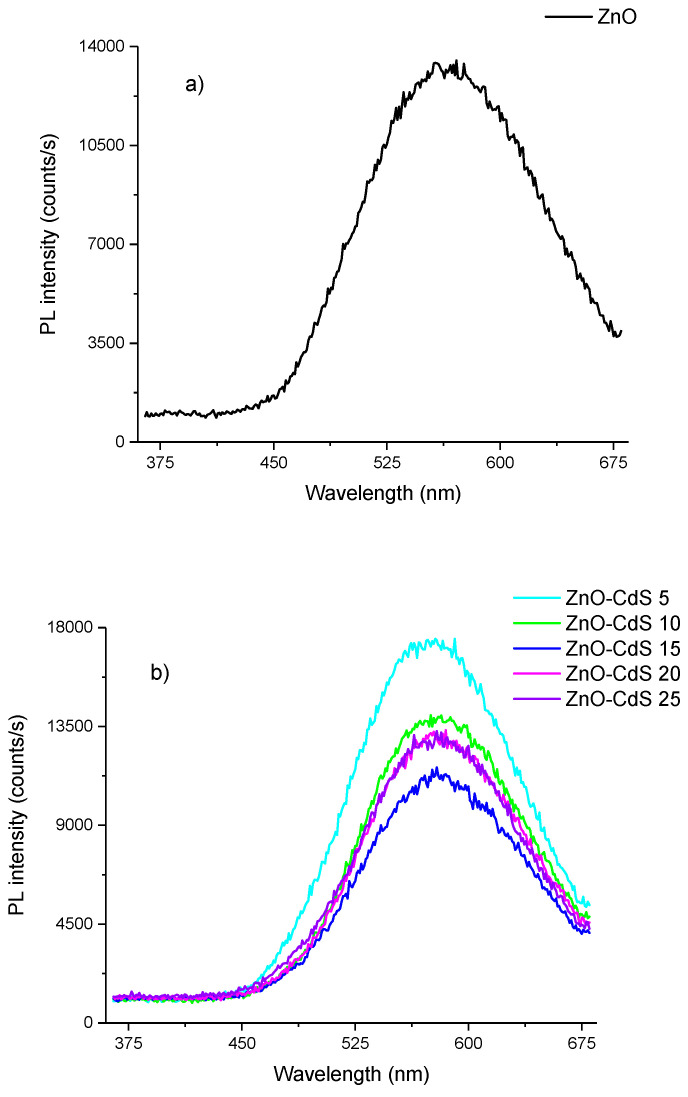
Photoluminescence spectra of (**a**) ZnO and (**b**) ZnO–CdS powders (λ_exc_ = 350 nm).

The optical properties of pristine ZnO and ZnO–CdS composite powders were analyzed by photoluminescence. Thus, all recorded PL spectra (Figure 5) disclose the characteristic ZnO emission bands: One very weak, barely observed, in the UV domain related to band to band transitions, peaked at~380 nm, and another broad intense in the visible region linked to defects with maximum at ~560 nm in the pristine compound and at~580 nm in the composite powders. 

Taking into account that the chemical precipitation is a wet chemical synthesis method in which the ZnO crystallites are formed by Zn(OH)_2_ dehydration, traces of this compound on the ZnO surface can lead to the quenching of the ZnO exciton emission [62]. Moreover, the incorporation of hydroxyl groups in the ZnO crystal lattice during the solution growth and to the oxygen defects can result in an increasing of the intensity of the visible emission [63,64]. Although various mechanisms responsible for the appearance of visible light are related to defects such as oxygen vacancy, zinc vacancy, interstitial oxygen, interstitial zinc, etc. [65], the origin of this emission is still on debate.

**Figure 6:**
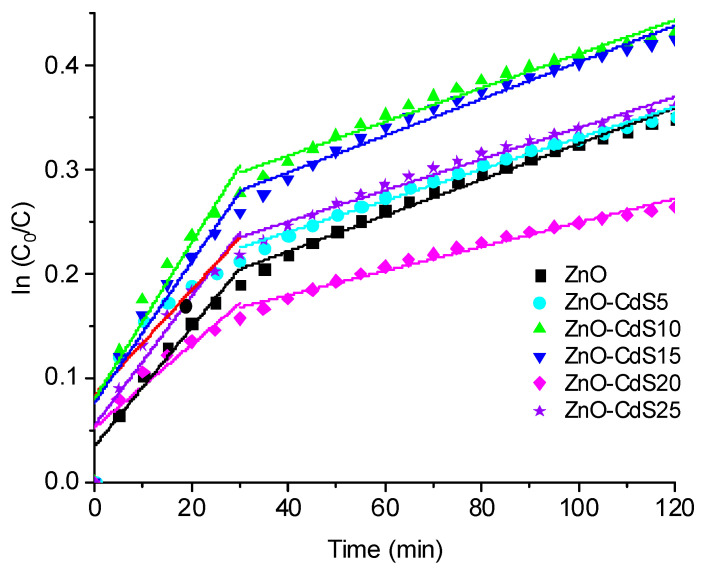
ln(*C*_0_/*C*) vs. time for MB discoloration in the presence of ZnO and ZnO–CdS powders. The linear fit of the data is also shown (the straight line).

Further, the photocatalytic activity of the pristine ZnO and ZnO–CdS composite powders was evaluated, the measurements being made on MB under UV illumination. Although, the discoloration rate was usually determined by a pseudo-first order linear regression corresponding to a simple first-order reaction model [66,67], the experimental curves shown in Figure 6 reveal two linear regions; the reaction rate constant values for each of them are given in Table 2.

The presence of these two regions can be interpreted if we consider that the MB photocatalytic discoloration involves the steps described below [66,67]:

(1) $$\mathrm{Organic}\;\mathrm{Dyes}\;(\mathrm{MB})\overset{}{⟶}\mathrm{Adsorption}\overset{k_{1}}{⟶}\mathrm{Intermediate}\;\mathrm{Product}\overset{k_{2}}{⟶}\mathrm{Colourless}\;\mathrm{Product}$$

The mechanism can be explained based on the peculiarity of our photocatalytic measurements. Generally, the investigation regarding the degradation of a dye in the presence of a photocatalyst in powder form is performed in an aqueous solution. As mentioned above, in this study, the photocatalytic tests were carried on samples obtained by immersing the powders disposed on glass substrates into MB aqueous solutions, further the powder impregnated with MB solution being dried in order to eliminate the water molecules, only the MB molecules remaining on the powder surface. In this way takes place the adsorption of MB molecules on the surface of the sample. Then, under UV irradiation, the MB discoloration leads to the formation of intermediate products, with a kinetic rate constant k_1_, subsequently these being transformed into colorless products, with a kinetic rate constant k_2_. Due to saturation with intermediate and colorless products of the powder surface, for all investigated samples, the k_2_ values are smaller than the k_1_ values. All ZnO–CdS composite powders (excepting the ZnO–CdS20) presented better photocatalytic activity than that of the pristine ZnO. Additionally, it has to be noticed that under UV light the MB discoloration does not occur in the absence of the photocatalyst powders, the measured reaction rate constant value was −4.509 × 10^−7^ min^−1^. 

**Figure 7:**
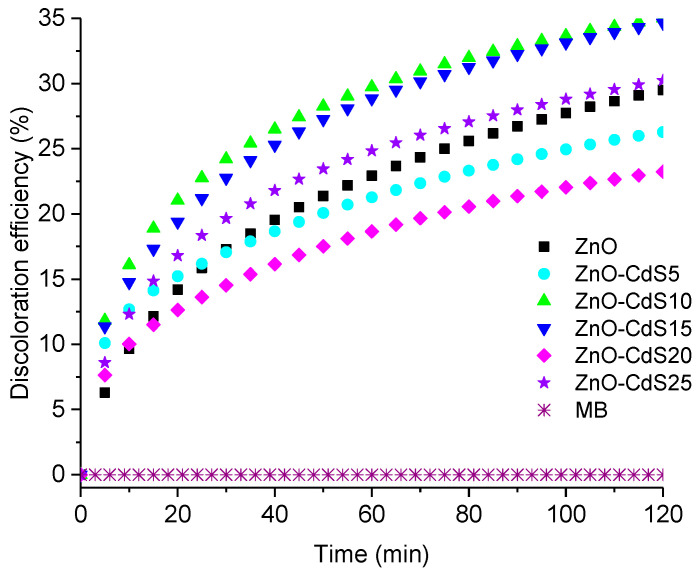
MB discoloration efficiency vs. time in the presence of ZnO and ZnO–CdS powders.

The MB discoloration efficiency of the ZnO–CdS composite powders is shown in Figure 7. According to [55,68], the mechanism involved in a dye discoloration in the presence of ZnO–CdS composites (the test was made in aqueous solution) can be described as follows: During the light irradiation, the band alignment between the two semiconductor components of the composites leads to the accumulation of the electrons in the ZnO conduction band and of the holes in the CdS valence band; the active species (hydroxyl radicals) generated by these charges being responsible for the dye degradation [21]. 

It is important to mention that when similar ZnO–CdS composites were used for the photodegradation of rhodamine B in aqueous solution, the presence of intrinsic defects and axial paramagnetic centers (so-called “shallow effective-mass donor” centers) can enhance their photocatalytic activity [53]. Hence, the presence of such defects and centers can explain why the correlation between the MB discoloration efficiency and the CdS amount from the composites is not a linear one, in our case; the best results being obtained for ZnO–CdS10 and ZnO–CdS15 powders.

**Figure 8:**
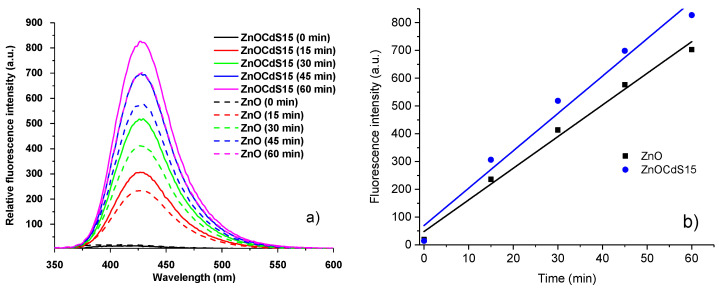
(**a**) Fluorescence spectra acquired in a solution containing TA (0.5 mM) and NaOH (2 mM), under UV light, at different irradiation times; (**b**) plot of the emission band intensity at 425 nm vs. irradiation time for ZnO and ZnO–CdS15 powders.

In order to detect the active species, OH•, involved in the MB discoloration, the photoluminescence investigations using TA as probe molecule were performed (Figure 8). Accordingly, under light irradiation, in aqueous solution, the reaction between TA and OH• generated by the photocatalyst powder leads to the formation of the TAOH, a highly fluorescent product, the intensity of its emission band being proportional to the OH• amount produced on the surface of the photocatalyst. The emission spectra acquired at different irradiation times on pristine ZnO and ZnO–CdS15 composite powders (Figure 8a) disclose that when the irradiation time increases, the intensity of the emission band centered at ~425 nm also increases, the generation rate of OH• being proportional to the irradiation time (Figure 8b); similar data being also recorded for the other composites. Moreover, the ZnO–CdS composite generates a larger OH• amount in comparison with the pristine ZnO, confirming that the presence of CdS along the ZnO as composite can improve the MB discoloration. 

Finally, the biological properties such as antioxidant capacity, antibacterial activity, and cytotoxicity of the ZnO, CdS, and ZnO–CdS composites were analyzed.

**Figure 9:**
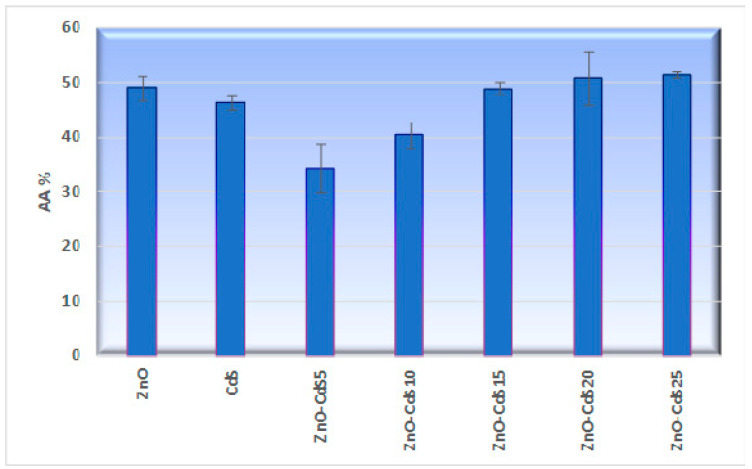
In vitro antioxidant capacity of ZnO, CdS, and ZnO–CdS powders.

The antioxidant capacity was assessed in vitro by chemiluminescence technique, the data being presented in Figure 9. The ZnO–CdS composite powders show medium antioxidant activity with values ranging between 35% and 52%, the parameter increasing with the increase of the CdS amount in the composites. An explanation for this result takes into account a recent study which provides evidence that the antioxidant activity of ZnO nanoparticles can be related to the transfer of electron density located at oxygen, to the odd electron located at outer orbits of oxygen in HO• and O_2_^•−^ species [69]. We assume that a similar process can take place in the case of our ZnO–CdS composite, where the electron density located at oxygen and sulfur can be transferred to the odd electron of activated oxygen species.

**Figure 10:**
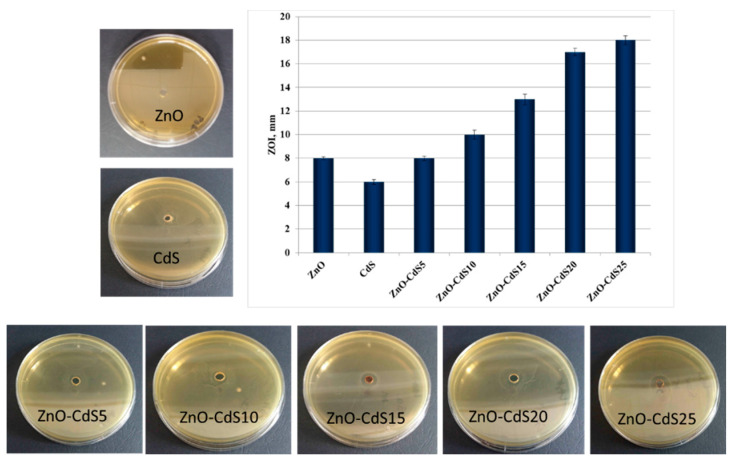
Antibacterial activity of ZnO, CdS, and ZnO–CdS powders against *Escherichia coli* ATCC 8738 and the corresponding zone of inhibition.

The antibacterial activity was evaluated against *Escherichia coli* Gram-negative bacteria, because this one has new forms which are responsible for various diseases [70]. Figure 10 displays the diameter of the inhibition zone for the *Escherichia coli* ATCC 8738 bacterial strain generated by each investigated powder, the ZOI diameter increasing also with the increase of the CdS amount in the composites. 

**Figure 11:**
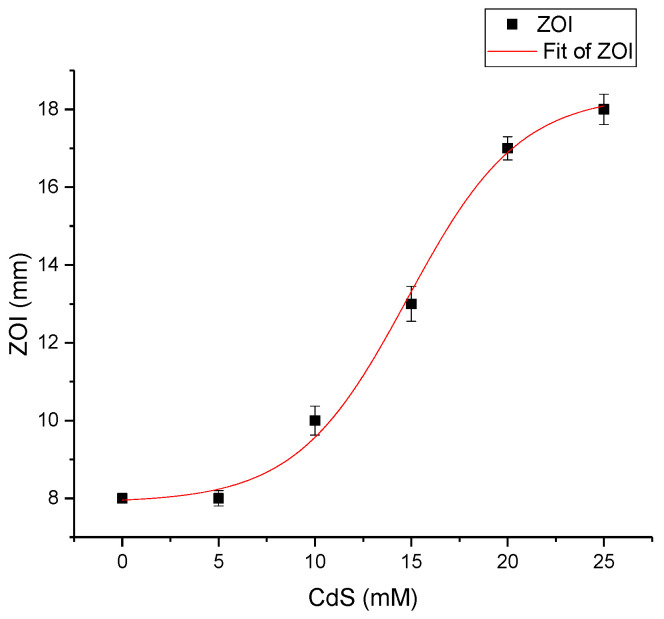
ZOI diameter (in mm) exhibited by the ZnO–CdS powders vs. CdS concentration.

Regarding the increase of ZOI diameter (Figure 11), this follows the equation: ZOI_CdS_ = 18.4 − 10.5/(1 + exp((C_CdS%_ − 3)/0.58)); (R^2^ = 0.99362). This dependence of ZOI with increasing CdS amount in the composites could help to estimate the diameter of the inhibition zone induced by a particular CdS amount in the ZnO–CdS powders.

For the pristine ZnO, the antibacterial mechanism can be described as: The structures interact directly with the *E. coli* cell walls, damaging the integrity of the bacterial cell, then they penetrate inside the cell causing membrane dysfunction and finally the death of the cell [71]. Comparatively, the pristine CdS induces less antibacterial activity due to its poor aqueous solubility [72]. By combining the both components into composites, the presence of ZnO improves the CdS water solubility, allowing a considerable better cellular uptake of the ZnO–CdS into the *E. coli* cells. It is known that, in the culture media, metal and metal oxide nanoparticles can release, from their surfaces, metallic positively charged ions, which can adhere by electrostatic forces to the negative charges of the peptidoglycan network from the bacterial cell wall, and in this way damaging it and altering the normal physiological processes of cells [73,74]. We suppose that a similar interaction can occur also between the ZnO–CdS particles and *E. coli* cell walls. On the other hand, the morphology of the composite materials can influence their antimicrobial properties. Thus, the particular flower morphology of the ZnO–CdS composite can help aid the destruction of the bacterial cell walls.

**Figure 12:**
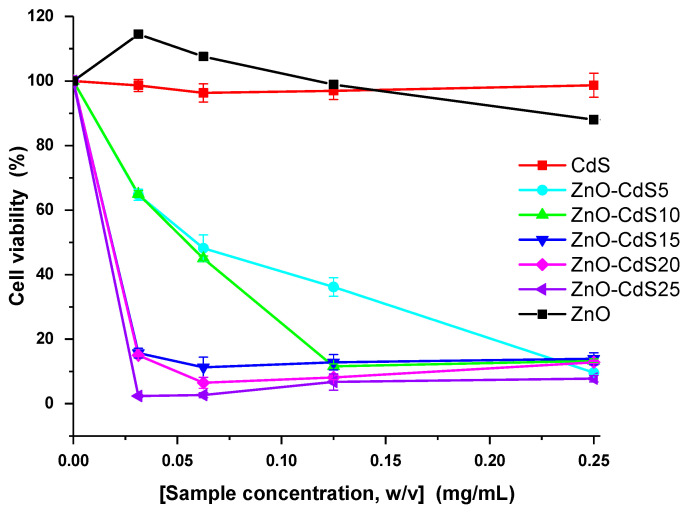
Cytotoxic effect of ZnO, CdS, and ZnO–CdS powders on BJ cells evaluated using MTT assay. Each value represents the mean ± SD of three experiments.

The cytotoxicity was tested against BJ cells for concentrations between 0.03 and 0.25 mg/mL; the results are given in Figure 12. ZnO and CdS in pristine form do not affect the viability of human fibroblast cells in the range of tested concentrations. For the ZnO–CdS5 composite, the viability of the cells decreases with the increase of the composite concentration, the viability decreasing to ~10% for the highest tested concentration. From the viability curve of this sample, the IC50 value was evaluated as being 57 μg/mL. The ZnO–CdS10 composite affects even more the viability of the cells having an IC50 of 50 μg/mL. The other composites, containing larger CdS amounts, were toxic even at the lowest tested concentrations, having IC50 of 17 μg/mL (ZnO–CdS15), 8.7 μg/mL (ZnO–CdS20), and 3 μg/mL (ZnO–CdS25). Although, the toxicity of ZnO–CdS composites increases with the CdS amount, taking into account the cytotoxic data, the ZnO–CdS5 and ZnO–CdS10 composites can be safely used, at concentrations less than 0.05 mg/mL.

Based on the photocatalytic tests and on the preliminary bioassay evaluation, it can be concluded that ZnO–CdS composites can find applications as photocatalytic and/or biocidal additives for photoactive coatings, paints, epoxy floors, etc. 

## 4. Conclusions

ZnO–CdS composite powders were prepared by chemical precipitation and were characterized from the morphological, structural, compositional, photocatalytical, and biological points of view. In pristine form, both components are featured by their typical structural and optical signatures: Hexagonal wurtzite phase and a band gap value at ~3.3 eV for ZnO and face-centered cubic phase and a band gap value at ~2.2 eV for CdS. The SEM images reveal that the ZnO is formed by spindle structures, the CdS by agglomerate nanoparticles, and the ZnO–CdS composites by flower-like structures with sizes depending on the CdS amount added during their chemical synthesis. The photocatalytic activity of the obtained samples was analyzed under UV irradiation using powders impregnated with MB solution; the tests confirmed that the presence of CdS along the ZnO can improve the MB discoloration. Biological properties such as antioxidant capacity, antibacterial activity, and cytotoxicity of ZnO, CdS, and ZnO–CdS composites were assessed. The ZnO–CdS composites present medium antioxidant effect, biocidal activity against *Escherichia coli,* and no toxicity (at concentrations less than 0.05 mg/mL for composites with a low CdS amount) for human fibroblast cells. Thus, using a simple wet synthesis method, which involves inexpensive equipment and raw readily available materials, ZnO–CdS composites can be prepared in large quantities and used as photocatalytic and/or biocidal additives for photoactive coatings, paints, epoxy floors, etc.; these providing a cleaner and healthier environment.

## Figures and Tables

**Table 1:** Chemical state concentrations obtained from the analysis of the extended XPS spectrum of the ZnO–CdS15 composite powder.

Chemical States	%
Zn2p^3/2^	41.3
O1s	45.6
C1s	6.0
Cd3d	3.2
S2p	3.9

**Table 2:** The reaction rate constants, k_1_ and k_2_.

Sample	k_1_ (min^−1^)	k_2_ (min^−1^)
ZnO	0.00568 ± 0.00022	0.00172 ± 0.00002
ZnO–CdS5	0.00503 ± 0.00045	0.00151 ± 0.00002
ZnO–CdS10	0.00746 ± 0.00046	0.00163 ± 0.00003
ZnO–CdS15	0.00673 ± 0.00039	0.00176 ± 0.00003
ZnO–CdS20	0.00397 ± 0.00029	0.00116 ± 0.00002
ZnO–CdS25	0.00617 ± 0.00033	0.00150 ± 0.00002
