# Correction: Zgura et al. Cytotoxicity, Antioxidant, Antibacterial, and Photocatalytic Activities of ZnO–CdS Powders. *Materials* 2020, *13,* 182

**DOI:** 10.3390/ma14247713

**Published:** 2021-12-14

**Authors:** Irina Zgura, Nicoleta Preda, Monica Enculescu, Lucian Diamandescu, Catalin Negrila, Mihaela Bacalum, Camelia Ungureanu, Marcela Elisabeta Barbinta-Patrascu

**Affiliations:** 1 National Institute of Materials Physics, Atomistilor 405A, 077125 Magurele, Romania; mdatcu@infim.ro (M.E.); ldiamandescu@gmail.com (L.D.); catalin.negrila@infim.ro (C.N.); 2 Department of Life and Environmental Physics, Horia Hulubei National Institute for Physics and Nuclear Engineering (IFIN-HH), Bucharest, 077125 Magurele, Romania; bmihaela@nipne.ro; 3 Faculty of Applied Chemistry and Materials Science, University “Politehnica” of Bucharest, 1-7, Polizu Street, 011061 Bucharest, Romania; ungureanucamelia@gmail.com; 4 Faculty of Physics, University of Bucharest, 405 Atomistilor Street, PO Box MG-11, Bucharest, 077125 Magurele, Romania; elipatras@gmail.com

## Error in Figure

In the original publication [1], there was a mistake in Figure 10 as published due to the duplication of a picture. The corrected Figure 10 appears below.

The authors apologize for any inconvenience caused and state that the scientific conclusions are unaffected. The original publication has also been updated.

## Figures and Tables

**Figure 10:**
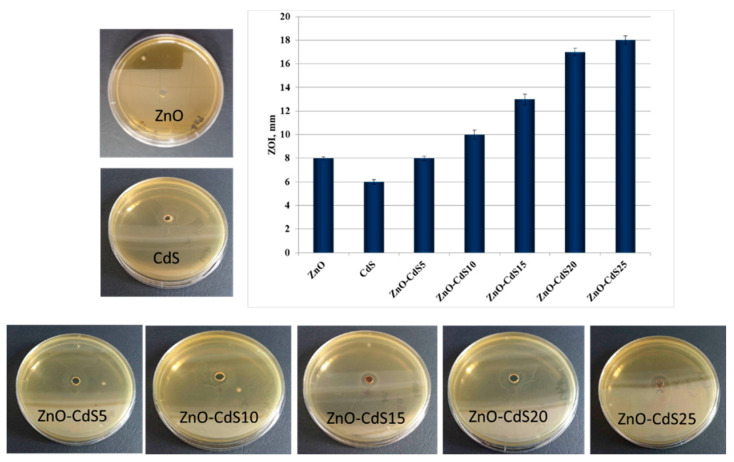
Antibacterial activity of ZnO, CdS, and ZnO–CdS powders against *Escherichia coli* ATCC 8738 and the corresponding zone of inhibition.
